# Differential Dermal Expression of *CCL17* and *CCL18* in Tuberculoid and Lepromatous Leprosy

**DOI:** 10.1371/journal.pntd.0003263

**Published:** 2014-11-20

**Authors:** William R. Berrington, Chhatra B. Kunwar, Kapil Neupane, Susan J. F. van den Eeden, James C. Vary, Glenna J. Peterson, Richard D. Wells, Annemieke Geluk, Deanna A. Hagge, Thomas R. Hawn

**Affiliations:** 1 University of Washington School of Medicine, Seattle, Washington, United States of America; 2 Mycobacterial Research Laboratory, Anandaban Hospital, Kathmandu, Nepal; 3 Department of Infectious Diseases, Leiden University Medical Center, Leiden, The Netherlands; University of Texas Medical Branch, United States of America

## Abstract

**Background:**

Leprosy is characterized by polar clinical, histologic and immunological presentations. Previous immunologic studies of leprosy polarity were limited by the repertoire of cytokines known at the time.

**Methodology:**

We used a candidate gene approach to measure mRNA levels in skin biopsies from leprosy lesions. mRNA from 24 chemokines and cytokines, and 6 immune cell type markers were measured from 85 Nepalese leprosy subjects. Selected findings were confirmed with immunohistochemistry.

**Principal Results:**

Expression of three soluble mediators (*CCL18*, *CCL17* and *IL-10*) and one macrophage cell type marker (*CD14*) was significantly elevated in lepromatous (*CCL18*, *IL-10* and *CD14*) or tuberculoid (*CCL17*) lesions. Higher CCL18 protein expression by immunohistochemistry and a trend in increased serum CCL18 in lepromatous lesions was observed. No cytokines were associated with erythema nodosum leprosum or Type I reversal reaction following multiple comparison correction. Hierarchical clustering suggested that *CCL18* was correlated with cell markers *CD209* and *CD14*, while neither *CCL17* nor *CCL18* were highly correlated with classical TH1 and TH2 cytokines.

**Conclusions:**

Our findings suggest that *CCL17* and *CCL18* dermal expression is associated with leprosy polarity.

## Author Summary

Leprosy presents with a polarized spectrum, with lepromatous leprosy having high bacillary numbers and TH2 dermal cytokines, versus tuberculoid leprosy showing very few bacilli and TH1 cytokines. The mechanism underlying this polarized presentation is largely unknown. In the following study, we isolated mRNA from skin biopsies from 85 individuals with leprosy and measured the expression of a panel of 24 cytokines and 6 cell markers. We found that three soluble mediators (CCL17, CCL18 and IL10) and one cell marker (*CD14*) were differentially expressed in leprosy dermal lesions. *CCL18* and *IL10* were more highly expressed within lepromatous lesions, and *CCL17* and *CD14* were more highly expressed within tuberculoid lesions. In addition, CCL18 protein expression was confirmed by immunostaining. *CCL17* and *CCL18*, were more strongly associated with leprosy polarity than traditional TH1 and TH2 cytokines. These data suggest that newer soluble chemokines may be important in leprosy pathogenesis and uncover a molecular signature of the two polar phenotypes of leprosy, which may be useful in future diagnostics.

## Introduction

Leprosy is a spectrum of illnesses involving the nerves, skin and upper airways of humans [1]. Depending on the host response to *Mycobacterium leprae*, the clinical presentation can vary widely between individuals [2]. Histologic features of these lesions can be quantitated on a 5 point classification system (Tuberculoid tuberculoid (TT), Borderline Tuberculoid (BT), Borderline Borderline (BB), Borderline lepromatous (BL), and Lepromatous Lepromatous (LL)) defined by Ridley and Jopling in 1966 which helps to define the polar spectrum found in clinical disease cause by *M leprae* [3]. In lepromatous leprosy (LL and BL), the clinical course and histologic features are distinguished by uncontrolled replication of the bacilli in dermal foamy macrophages and poor granuloma formation. In tuberculoid leprosy (BT and TT), the skin lesions are characterized by well-demarcated granulomatous inflammation within nerves and skin, CD4 and CD8 T cell dermal infiltration, and little evidence of bacilli within lesions.

Early studies of leprosy intradermal cytokine expression determined that leprosy polarity is associated with TH2 cytokines *IL4*, *IL5*, *IL10* in lepromatous lesions, and TH1 polar cytokines, *IFNG*, *IL2*, in tuberculoid lesions [4]. In addition, CD4 T-cell frequency was increased in the tuberculoid lesions compared to the lepromatous lesions [5]. Since these early seminal studies, many additional cytokines and chemokines have been identified but not analyzed in leprosy lesions. Genome-wide transcriptional profiling has also been used to define cytokine mRNA levels within lesions [6]. The sample size of polar subgroups was low in this study and precluded a full comparison of the tuberculoid and lepromatous groups. To date, a detailed study of leprosy using larger sample sizes and surveying a broad, current repertoire of cytokines and chemokines has not been performed.

Differentiation and polarization of T cell subsets is characterized by a complex network of cytokines and chemokines [7], [8]. Initially, T-cell subsets were defined by their release of specific cytokines; TH1 associated with *IFNG* and *IL2* and TH2 subset associated with release of *IL4*, *IL5*, and *IL10*. Since these influential studies, the number of cytokines that defined T cell subsets have been expanded to include TH17, TH22, and Tregs [8]. *CCL17* and *CCL18* are chemokines important in T cell mediated reactions [9]–[15]. *CCL17* is secreted by alternatively activated macrophages [16] and is markedly elevated in patients with allergic atopic dermatitis who have a TH2 dominance [15]. In murine studies, *CCL17* has been implicated in both TH1 and TH2 responses [15]–[19]. *CCL17* is increased in dendritic cells activated with *M. tuberculosis* [20]. *CCL18*, is elevated in atopic dermatitis [21] and also is secreted by dendritic cells upon recognition of MTB by innate immune cells [22]. Additionally, *CCL18* has been implicated in differentiation of macrophages into an alternatively activated phenotype [23].

In the following study, we measured the mRNA levels of 24 soluble cytokines and 6 cell specific markers in 82 individuals with biopsy-confirmed polar leprosy with the goal to better characterize and define the immune responses in individuals with leprosy phenotypes. Herein we show that two chemokines, *CCL17* and *CCL18*, more accurately define leprosy polarity than traditional TH1 and TH2 cytokines.

## Methods

### Ethics Statement

All human subjects provided informed consent to the procedures below. No children were enrolled in the study. Both written and oral informed consent was given due to the high rates of illiteracy. Subjects that were unable to read and write provided a thumbprint as proof of consent, while those that could read and write provided a signature. All informed consent documents and procedures were approved by the University of Washington Institutional Review Boards per the US Department of Health and Human Services Guidelines, The Medical Ethics Committee of Leiden University Medical Center, and Nepal Health Research Council (NHRC).

### Human Subjects and Study Design

Dermal biopsies were obtained from patients at Anandaban Hospital in Kathmandu, Nepal. These cases comprised more than 8 different ethnic and religious groups included Vaishya, Chhetri, Brahmin, and Sudra (Table 1). Eighty five patients were enrolled based on clinical presentation with the leprosy diagnosis confirmed by skin slit smears and biopsy. One of the biopsy samples was used for histological diagnosis and the other for isolation of mRNA. Leprosy class was determined by Ridley Jopling (RJ) classification [3] after the biopsies were fixed, mounted in paraffin, and stained with both Fite stain and hematoxylin and eosin for viewing under conventional light microscopy by experienced leprosy pathologists at the Schieffelin Institute of Health – Research & Leprosy Centre, Karigiri, Tamil Nadu, India. mRNA levels from dermal biopsies were measured by RT-PCR with a BioMark Fluidigm platform which included 38 with tuberculoid (borderline tuberculoid (BT) and tuberculoid (TT)), 3 with borderline borderline (BB), and 44 with lepromatous leprosy (borderline lepromatous (BL) and lepromatous (LL)) (Table 1). Although Bacillus Calmette–Guérin (BCG) vaccination for the participants was not recorded, it is routine for individuals in Nepal to get vaccinated as an infant in Nepal. Routine population-wide vaccination began in 1966 and therefore individuals born before then may have not received the BCG vaccine as an infant [24]. The biopsies were obtained in a leprosy tertiary care center, where a significant number of people diagnosed with leprosy reactive states are referred. 48 patients out of 85 undergoing analysis for mRNA levels had reactions at the time of biopsy, including the three diagnosed with BB disease. 45 of these patients were included in the polarity analysis and shown in Table 1. For ELISA studies 20 additional, newly diagnosed patients and 6 control individuals from the same region (endemic controls: EC) had serum collected. In addition, skin biopsies were performed as above to determine RJ classification.

**Table 1:** Leprosy biopsy demographics.

		Tuberculoid		Lepromatous		pvalue
Age	(Mean±SD)	35.7	±15.6	37.3	±14.4	0.65
Sex	male (%)	24	(63%)	39	(89%)	
	female (%)	14	(37%)	5	(11%)	0.01
Ethnicity	Vaishya	8	(21%)	17	(39%)	
	Chhetri	8	(21%)	7	(16%)	
	Other	8	(21%)	7	(16%)	
	Brahmin	7	(18%)	7	(16%)	
	Sudra	7	(18%)	5	(11%)	
	Indian	0	(0%)	1	(2%)	0.51
Reaction	RR^1^	16	(42%)	20	(45%)	0.76
	ENL^2^	0	(0%)	9	(20%)	<0.01

1 Reversal Reaction,

2 Erythema nodosum leprosum.

### RNA Isolation and RT-PCR

Half of each biopsy sample was macerated in RNA*later* (Invitrogen, Carlsbad, CA) preservative and stored at −20°C for later processing. Samples were homogenized with a high shear homogenizer (OMNI international, Kennesaw GA) using disposable tips. Total RNA was isolated using RNeasy mini columns and cDNA was made using Applied Biosystems high capacity cDNA reverse transcriptase kits (Foster City CA). RT-PCR was performed with PrimeTime primer probe sets from Integrated DNA Technologies (Corallville, IA) (Table 1) and Taqman (Life Technologies) for genes *CD1a* (catalog number Hs00233332_m1) using the Fluidigm 48×48 dynamic array platform. Briefly 2.5 ng of DNA was preamplified in a 5 ul reaction with 25 nM concentration of all primers and 12.5 nM concentration of all probes (For list see Supplemental Table S1) and amplified for 15 cycles with a 30 sec denaturation step at 98°C and 4 minutes at 60°C. The pre-amplified reaction was added to the Fluidigm 48×48 dynamic array platform for each individual diluted in 2× mastermix buffer. 10× IDT PrimeTime gene expression assays were added to the assay portion of the Fluidigm chip and amplified for an additional 40× cycles in the Fluidigm assay chip. To verify that the Fluidigm assay was accurate, for a set of probes (*CCL17*, *CCL18*, and *CD1a*) we did single 20 ul RT PCR assays. Both singleplex assays and Fluidigm chip assays values were corrected by the *GAPDH* values to control for variability in biopsy size, mRNA yield, and cellularity within biopsy samples. Single assay RTPCR and Fluidigm values had R^2^ values of approximately 0.7–0.9 values (Supplemental Figure S1).

### Immunohistochemistry

4 um paraffin sections were deparaffinized and rehydrated with heat-mediated antigen retrieval performed in citrate buffer (pH 6). Slides were blocked with 2.5% normal horse serum, incubated with anti-CCL18 primary antibody (Peprotech, Catalog # 500-P108) and anti-CCL17 (RND Systems, Catalog #AF364) overnight at 4°C followed by ImmPress rabbit HRP (CCL18) and ImmPress goat HRP (CCL17) (Vector Laboratories, Burlingame CA). Slides were developed with QuantoDAB (Fisher Scientific) and counter-stained with hematoxylin. Stained tissue biopsies were scored by a dermatologist blinded to the subject's leprosy classification. Sections were surveyed on low power, and representative areas showing dermal or subcutaneous inflammation on each specimen were identified. Several representative 40× fields were assessed in each specimen and scored as 0 for <1%, 1+ for 1–10%, 2+ for 10–20%, or 3+ for >20% cells staining for CCL18.

*ELISA*. Six (6) control and 20 individuals with documented leprosy had sera collected and assayed for CCL17 and CCL18 protein levels by sandwich ELISA (RND biosystems), per manufacturers protocol.

### Statistics

The Mann-Whitney U-test was used to make comparisons of the cytokine production between groups, as small sample sizes precluded an assumption of normal distribution. Two-sided testing was used for all comparisons to evaluate statistical significance. A P value of ≤0.05 was considered significant in initial analysis. Bonferroni corrections for multiple comparisons were added as described. Statistics were calculated with Stata software (version 11.2). For dermal expression correlation the non-parametric Spearman's rank correlation test *rho* (ρ) statistic was used for correlation coefficient, and was generated using R program version 3.0.1 (R: A Language and Environment for Statistical Computing, Vienna Austria). For iterative analysis, we used Stata 11.0 random number generator to randomly assign discovery and validation cohorts on 40 successive iterations and used Mann-Whitney U test for significance. For iterative analysis P values were not adjusted for multiple comparisons; P values of less than 0.05 were considered significant. Hierarchical clustering of the Spearman's ρ statistics was performed in R program using the complete-linkage method [25] which clusters individual tests based on the maximum distance between tests [26]. Graphical representation of clustering was displayed using corrplot package by Teiyun Wei in R program [27], [28].

### Accession Numbers (RefSeq)

The following HGNC genes and corresponding refseq accession numbers were used in the paper: *CCL1*, NM_002981; *CCL17*, NM_002987; *CCL18*, NM_002988; *CCL2*, NM_002982; *CD14*, NM_000591; *CD1A*, NM_001763; *CD209*, NM_021155; *CD22*, NM_001771; *CD3D*, NM_000732; *FOXP3*, NM_014009; *GAPDH*, NM_002046; *IFNA1*, NM_024013; *IFNA8*, NM_002170; *IFNB1*, NM_002176, *IFNG*, NM_000619; *IL10*, NM_000572; *IL12A* NM_000882; *IL12B* NM_002187; *IL13*, NM_002188; *IL17A*, NM_002190; *IL18*, NM_001562; *IL1B*, NM_000576; *IL1RN*, NM_000577; *IL21*, NM_021803; *IL22*, NM_020525; *IL23A*, NM_016584; *IL27*, NM_145659; *IL4*, NM_000589; *IL6*, NM_000600; *TNF*, NM_000594; *IFNL1*, NM_172140

## Results

### Cytokine/Chemokine Expression in Dermal Lesions from Leprosy Patients

We examined whether skin mRNA levels of candidate immune genes differed in individuals with tuberculoid (TT/BT, n = 38) and lepromatous (BL/LL, n = 44) leprosy. The cohort included Nepalese adults evaluated at a tertiary care center for leprosy (Table 1). Skin biopsies were analyzed for mRNA levels of TH1 and TH2 chemokines/cytokines, type I, II and III interferons, other T-cell associated cytokines, *IL12* family members, *IL1β* family members, cellular markers, and house-keeping genes (for complete list see Table S1). Of the 24 soluble mediators of inflammation that were measured, 7 showed significant differences between lepromatous and tuberculoid leprosy (*CCL2*, *CCL17*, *CCL18*, *IFNA1*, *IL10*, *IL22*, and *TNF*) (Table 2). *CCL2*, *CCL18*, *IL10* expression was higher in lepromatous lesions, while *CCL17*, *IFNA1*, *TNF*, and *IL22* were more highly expressed in tuberculoid lesions. In addition, median values for the prototypic TH1 cytokine, *IFNG*, were higher in tuberculoid lesions compared to lepromatous, but it was not statistically significant (median values = 0.212 vs 0.015, P = 0.103). Only *CCL17*, *CCL18* and *IL-10* remained significant after adjustment for multiple comparisons using Bonferroni correction. Stratification of samples by type 1 reaction for leprosy reactive state did not alter the associations observed for *CCL17* and *CCL18* (For P values see below). In order to adjust for age we analyzed *CCL17* and *CCL18* associations with leprosy phenotype in patients born before 1966 (cutoff age of 45). We found that the majority of the association of CCL17 and CCL18 expression was seen in individuals less than 45 (age >45 n = 26: CCL17, P = 0.09; CCL18, P = 1.4×10^−3^), (age <45, n = 56; CCL17, P = 8.2×10^−4^; CCL18, P = 5.7×10^−5^).

**Table 2:** Cytokine expression normalized by GAPDH in dermal leprosy lesions.

	TT+BT		LL+BL			
Cytokine	median	75 (%)	median	75 (%)	pValue	pValue (adj)
CCL1	4.50E-05	5.20E-04	0.000	1.41E-04	0.212	5.081
CCL2*	0.033	0.161	0.146	0.372	0.009	0.218
**CCL17**	**0.024**	**0.067**	**0.005**	**0.011**	**2.36E-04**	**0.006**
**CCL18**	**0.041**	**0.089**	**0.309**	**0.493**	**3.00E-07**	**7.20E-06**
IFNA1*	0.009	0.013	0.006	0.009	0.008	0.182
IFNA8	0.045	0.059	0.034	0.064	0.503	12.067
IFNB1	0.052	0.067	0.040	0.072	0.357	8.571
IFNG	0.212	0.479	0.015	0.228	0.103	2.478
**IL10**	**0.011**	**0.028**	**0.044**	**0.085**	**0.001**	**0.022**
IL12a	0.000	1.22E-04	0.000	2.28E-04	0.664	15.928
IL12b	0.001	0.003	0.001	0.003	0.615	14.758
IL13	0.000	0.000	0.000	0.000	0.326	7.831
IL17a	0.000	1.73E-04	0.000	5.10E-05	0.275	6.612
IL18	0.092	0.130	0.078	0.104	0.204	4.904
IL1b	0.022	0.048	0.018	0.040	0.270	6.481
IL1ra	2.497	3.804	1.766	3.385	0.787	18.898
IL21	0.000	2.36E-04	0.000	1.26E-04	0.645	15.470
IL22*	0.000	5.05E-06	0.000	0.000	0.047	1.129
IL23	0.011	0.025	0.007	0.014	0.091	2.193
IL27	0.000	4.96E-05	0.000	1.13E-04	0.729	17.494
IL29 (IFNL1)	0.000	0.000	0.000	7.42E-06	0.697	16.739
IL4	0.000	0.000	0.000	0.000	0.484	11.611
IL6	0.006	0.015	0.009	0.018	0.461	11.069
TNF*	0.032	0.047	0.023	0.032	0.028	0.675

TT+BT is tuberculoid polar leprosy, LL+BL is lepromatous polar leprosy. Values with Ct Values greater than 40 were given a value of zero and included in the statistical analysis to determine medians and 75^th^ percentile values. P values were calculated after Mann-Whitney analysis. Adjusted p values are the original p values corrected for multiple comparisons by traditional Bonferroni correction.

In bold are cytokines that were significant following Bonferroni correction.

*Represents cytokines that were significant by Mann-Whitney, but failed multiple comparisons corrections.

To further adjust for multiple comparisons, we randomly arranged our data into a discovery (n≈20) and validation cohort (n≈20) and ran the analyses on 40 separate sample iterations. Increased *CCL18* levels were associated with lepromatous leprosy in randomly generated discovery and validation cohorts in 40/40 (100%) of the iterations, while increased *CCL17* levels were associated with tuberculoid lesions in 29/40 (73%). On the other hand, *IL10* expression, only distinguished polarity in 18/40 (45%) of the iterations.

**Figure 1:**
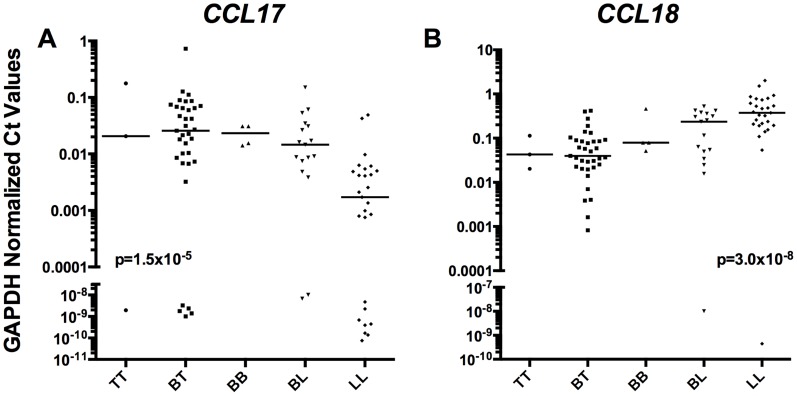
*CCL17* and *CCL18* by Ridley Jopling classification. Relative tissue mRNA levels of *CCL17* (A) and *CCL18* (B) normalized to GAPDH were arranged in order of specific leprosy classification and analyzed for significance by non-parametric trend test with variables TT, BT, BB, BL, and LL treated as increasing categorical variables, p values displayed. Lines are medians. A value of 40 was given to undetectable Ct Values in order to graphically represent on log scale.

Both *CCL17* (Figure 1A) and *CCL18* (Figure 1B) expression were highly significant when analyzed in rank order based on Ridley-Jopling classification (non-parametric trend test, P = 1.5×10^−5^ and 3.0×10^−8^, respectively). Together, these data suggest that both *CCL17* and *CCL18* dermal mRNA levels are associated with tuberculoid and lepromatous leprosy, respectively and are more strongly associated with polarity than common TH1/TH2 markers typically used to characterize leprosy lesions.

### Cellular Marker Expression in Leprosy Skin Biopsies

Next we analyzed the lesions for mRNA expression of markers known to distinguish innate immune cells from B and T cells (*CD14*-macrophage, *CD209*-dendritic cell, *CD1a*-dendritic and Langerhans cells, *CD22*-B cell, *CD3d*-T cells, *FoxP3*-T-Regulatory cells) (Table 3). We measured cell specific mRNA to both determine which cell types were predominant in the lesions and whether certain mediators were associated with a particular cell type. *CD14* and *CD209* were more highly expressed in lepromatous lesions, while *CD1a* had higher expression in tuberculoid lesions. *CD14* remained significant after corrections for multiple comparisons with Bonferroni adjustment. These data confirm that macrophage infiltration is a characteristic of lepromatous lesions.

**Table 3:** Cell marker expression in dermal leprosy lesions.

	TT+BT^1^		LL+BL^2^			
Cytokine	median	75 (%)	median	75 (%)	pValue^3^	pValue (adj)^4^
**CD14**	**4.50E-05**	**5.20E-04**	**0.000**	**1.41E-04**	**1.57E-04**	**0.001**
CD1a*	0.033	0.161	0.146	0.372	0.011	0.067
CD209*	0.024	0.067	0.005	0.011	0.031	0.186
CD22	0.041	0.089	0.309	0.493	0.748	4.490
CD3d	0.009	0.013	0.006	0.009	0.088	0.528
FoxP3	0.045	0.059	0.034	0.064	0.214	1.282

1 Tuberculoid polar leprosy,

2 lepromatous polar leprosy. Values of 0.000 are Ct values less than 1/2^40^.

3 P values were calculated by Mann-Whitney tests. Both median and 75^th^ percentile (75(%)) are depicted.

4 Adjusted p values are the original p values corrected for multiple comparisons by traditional Bonferroni correction.

In bold are cytokines that were significant following Bonferroni correction.

*represents cytokines that were significant by Mann-Whitney, but failed multiple comparisons corrections.

### Analysis of Cytokine and Cell Marker Expression in Leprosy Reactive States

**Figure 2:**
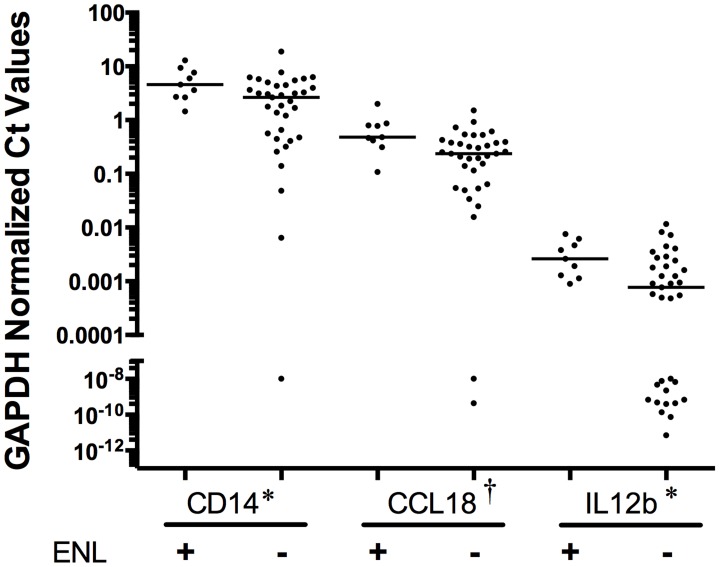
Dermal levels of *CD14*, *CCL18*, and *IL12a* mRNA in patients with ENL compared to those without. Expression levels of *CD14*, *CCL18*, and *IL12a* (normalized by GAPDH expression) were compared in patients with ENL (n = 9) versus those in the lepromatous pole without ENL (n = 35). Lines are medians. A value of 40 was given to undetectable Ct values in order to graphically represent on log scale. P values were †<0.01, *<0.05, based on Mann-Whitney analysis.

**Figure 3:**
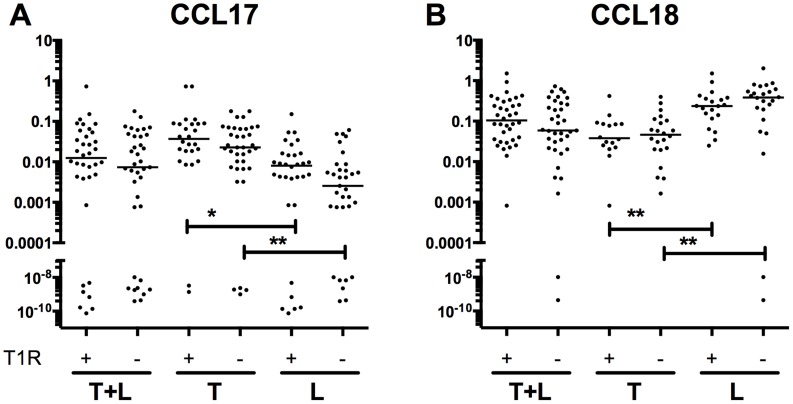
CCL17 and CCL18 mRNA are not associated with reactive state. CCL17 and CCL18 mRNA levels (normalized by GAPDH expression) were compared in LL+BL versus TT+BT patients with T1R (L pole (n = 16), T pole (n = 20)) versus those without T1R (L pole (n = 17), T pole (n = 22)). Lines are medians. A value of 40 was given to undetectable Ct Values in order to graphically represent on log scale. P values were **<0.01, *<0.05, based on Mann-Whitney analysis.

We next examined whether cytokine and cellular markers were significantly different between clinical leprosy reactive states. We analyzed expression levels in BL/LL patients with type 2 ENL reactions (n = 9) compared to those without type 2 ENL reactions (n = 35) and determined that transcripts from mediators *CCL18*, *IL12b*, and cell marker CD14 were significantly elevated in lesion samples with ENL (Figure 2). However, all of these failed to reach significance when adjusted for multiple comparisons. Next we examined mRNA levels in patients from whom biopsies were obtained with ongoing type 1 reversal reaction (n = 36, 42% of the tuberculoid patients and 45% of lepromatous patients) versus those who had no reversal reaction (n = 46, 58% of tuberculoid patients and 55% of lepromatous patients). No marker distinguished these two clinical phenotypes. In addition we performed subgroup analysis of mRNA level differences between patients with type 1 reversal reaction and those without type 1 reversal reaction limited to individuals with either tuberculoid leprosy or lepromatous leprosy, and also saw no differences in the cytokines transcript levels examined. Cytokine levels of *CCL17* (Figure 3A) and *CCL18* (Figure 3B) were significantly different between lepromatous and tuberculoid lesions regardless of the type 1 reactive state (*CCL17* with T1R P = 0.0038, without T1R P = 0.011, *CCL18* with T1R p = 3.6×10^−4^, without T1R P = 2.6×10^−4^).

### Increased CCL18 Protein Staining in Lepromatous Lesions

**Figure 4:**
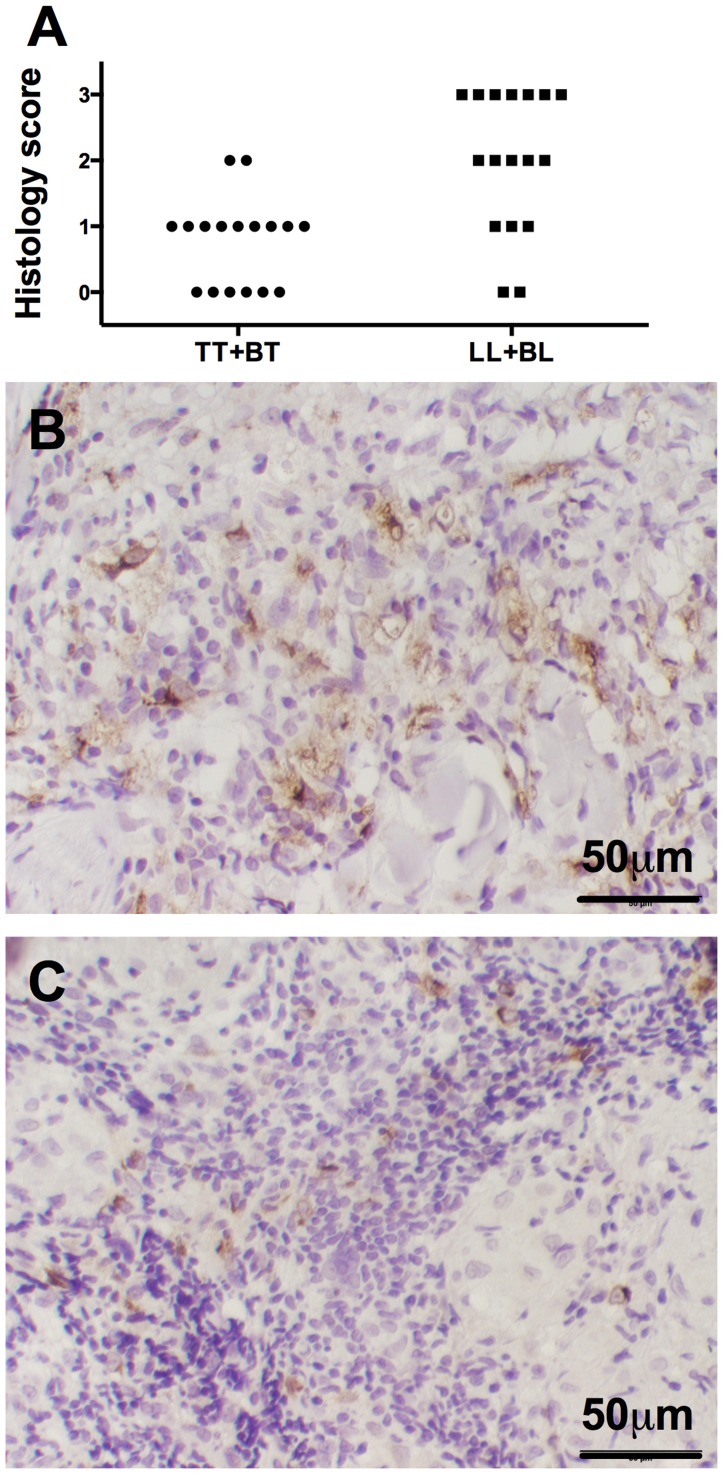
CCL18 immunohistochemistry in leprosy dermal biopsies. A) Semi-quantitative score of CCL18 immunohistochemical staining in LL+BL (n = 17) and TT+BT (n = 17) lesions. P<0.01 by non-parametric Mann-Whitney analysis. Representative pictures from B) LL+BL (score = 3) and C) TT+BT (score = 1) individuals are depicted.

Next we examined *CCL17* and *CCL18* protein expression within dermal lesions (n = 17 LL+BL and 17 TT+BT) using antibodies directed to human *CCL17* and *CCL18*. Control staining using secondary antibodies showed no immunoreactivity. We were unable to visualize *CCL17* staining above background levels despite using multiple antibody dilutions. *CCL18* stained cells were detected, primarily in the cytoplasm of large polygonal cells within the dermis and subcutaneous tissues most consistent with the monocyte-derived histiocytes typically found in such inflammation. Lesions stained with *CCL18* antibodies were scored semi-quantitatively for percent of immunoreactive cells (Figure 4A). More *CCL18* staining was seen in LL+BL lesions (Figure 4B) compared to TT+BT lesions (Figure 4C) (non-parametric Mann-Whitney test P<0.01).

### Serum CCL17 and CCL18 Protein Levels in Patients with Clinical Leprosy

**Figure 5:**
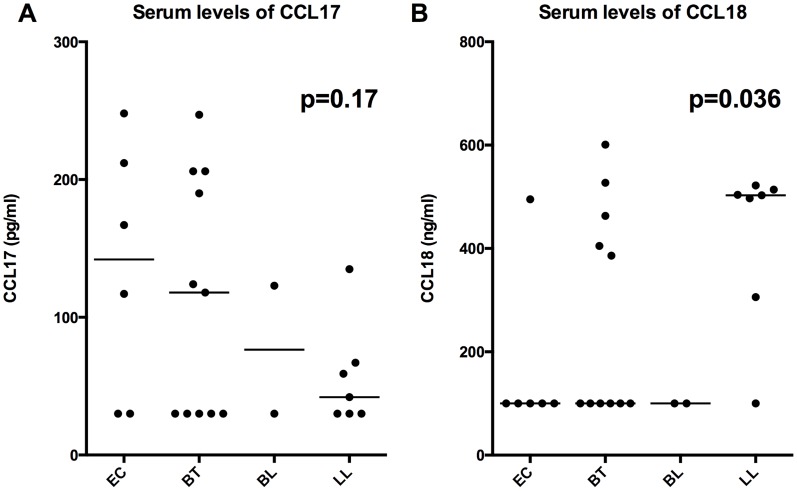
CCL17 and CCL18 serum levels. Serum levels of CCL17 and CCL18 protein were measured in leprosy patients with lesions defined by RJ classification (n = 20) compared to EC (n = 6). P values by non-parametric trend test are depicted. EC (patients without leprosy) were given a categorical value of 0, while those with leprosy were given increasing categorical values as progressing to LL pole. Lines depict median values.

Next the serum levels of *CCL17* and *CCL18* were compared in 6 EC (5 from Nepal and one from non-endemic area) patients without leprosy, versus 20 patients with leprosy (11 with BT, 2 with BL, and 7 with LL by RJ classification as determined by biopsy). Patients with lepromatous leprosy had a non-significant trend (p = 0.16) of decreasing *CCL17* levels (Figure 5A) and a significant trend (p = 0.036) of increasing *CCL18* (Figure 5B) levels in the serum.

### Hierarchical Clustering Determines Distinct Signatures Associated with CCL18 but Not CCL17 Expression in Dermal Biopsies

**Figure 6:**
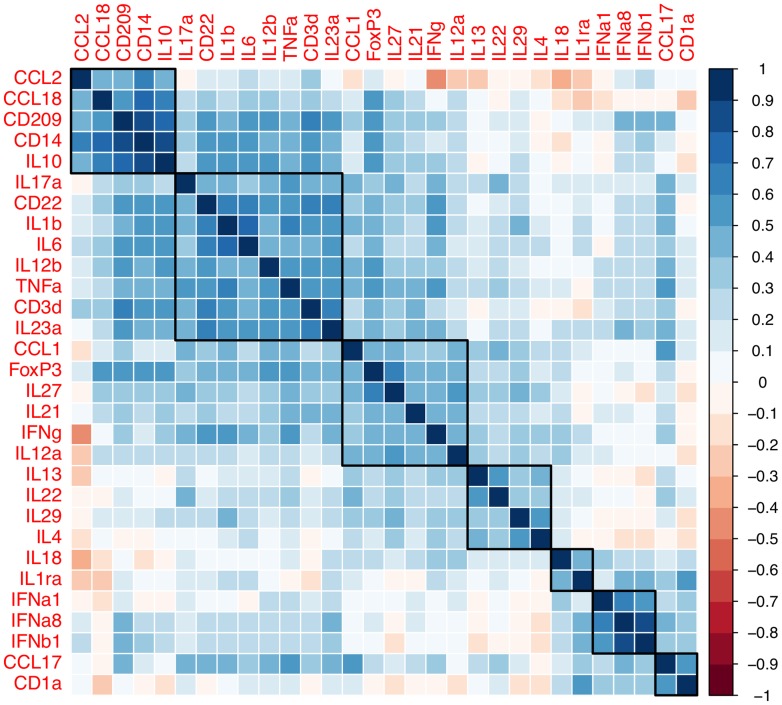
Correlation of dermal mRNA in cutaneous leprosy lesions. Values from correlation tests (Spearman's *rho* statistics) are plotted from GAPDH normalized mRNA expression levels. Groups of probes were associated by hierarchical clustering using the complete-linkage clustering method (see methods).

We next examined which cytokines and chemokines, correlated and clustered with cell marker levels (Figure 6). Our analyses revealed that *CCL18* and *IL10* expression were associated with *CD209* and *CD14* expression (Spearman's *rho* = 0.13 to 0.87), dendritic and macrophage markers respectively. In contrast, *CCL17* expression was poorly correlated to all cell type markers except the dendritic and Langerhans marker, *CD1a* (*rho* = 0.53). Hierarchical clustering identified 5–7 main groups that had similar signatures (Figure 6): *CCL18* group (*IL10*, *CD209*, *CD14*, and *CCL2*), *TNF* group (*IL17a*, *CD22*, *IL1β*, *IL6*, *IL12b*, *CD3d*, and *IL23a*), *IFNG* group (*CCL1*, *FoxP3*, *IL27*, *IL21*, and *IL12a*), *IL4* group (*IL13*, *IL22*, and *IL29* (*IFNλ1)*) and type I interferons (*IFNA1*, *IFNA8*, and *IFNB1*). *CCL17* and *CD1a* also tended to cluster with type I interferons. These data suggest that within leprosy lesions *CCL18* and *CCL17* expression may be associated with specific innate immune cells rather than TH1 and TH2 T-cell cytokines traditionally thought to define leprosy (*IFNG* and *IL4*).

## Discussion

The primary finding of our study is that dermal mRNA levels of *CCL17* and *CCL18*, two chemokines important in the development of a TH2 T-cell response, are associated with tuberculoid and lepromatous leprosy respectively. We also found that classical markers for leprosy and T-cell subset polarity, *IL4* and *IFNG*, were inferior in distinguishing polar leprosy compared to *CCL17* and *CCL18*. We also confirm an association of increased expression of *IL10* within lepromatous lesions.

Our findings highlight an association of increased dermal expression of *CCL17* in the skin of patients with tuberculoid leprosy. The mechanism of how *CCL17* is increased in the dermal lesions of patients, who have been traditionally thought to have TH1 polar disease, is largely unknown, but there may be many possible explanations. First, increases in *CCL17* expression may arise by direct stimulation of resident cells by mycobacterial antigens. Previous data suggest that *CCL17* secretion from DCs is associated with increased TH1 responses [17], [18]. However, other papers suggest that *CCL17* is associated with TH2 responses [13]–[15], [19]. Direct stimulation of *CCL17* mRNA expression by mycobacterial antigens would suggest that the response to *M. leprae* in patients with TT and BT disease may have more of a TH2 polarity than had been previously thought. To support this, there have been reports of increased *CCL17* expression in dendritic cells stimulated with *M. tuberculosis* in mice [20], but this has not been shown in humans. Second, elevated dermal expression of *CCL17* could occur following recruitment of a cell type that constitutively expresses *CCL17* in patients with tuberculoid leprosy. Third, chronic *M. leprae* exposure could promote differentiation of resident myeloid cells into *CCL17*-secreting cells that are more apt to control *M. leprae* replication. These second and third mechanisms would require there being significant differences in innate *M. leprae* detection between individuals with tuberculoid and lepromatous leprosy leading to either differences in cellular differentiation, or differences in the release of soluble mediators by resident cells that would influence differentiation or recruitment of new cells to lesions. Recent data suggest that NOD2 regulates *M. leprae* induced differentiation of myeloid precursors into cells associated with tuberculoid lesions [29]. Whether the immune cell associated with tuberculoid lesions (thought to be *CD1b*(+)) more actively produce *CCL17* is currently unknown. Ultimately the mechanism that underlies the association of increased expression of *CCL17* in tuberculoid lesions will need to be elucidated in future research.

The mechanism behind the association of increased *CCL18* expression in lepromatous lesions is similarly unclear. Our clustering data suggest that *CCL18* expression is associated with the expression patterns of *IL10*, *CD209* and *CD14* in lepromatous lesions, possibly implying that recruitment or differentiation of a specific cell type may be one mechanism to support the increased expression of *CCL18*. In support of this hypothesis, studies have described *CD14*+ and *CD209*+ monocyte-derived skin antigen presenting cells that have an immature or tolerant phenotype in normal human skin [30], [31] and have been associated with the production of *CCL18* [32]. Whether these cells exist in lepromatous leprosy lesions, is not known. Mycobacterial antigens present in leprosy lesions could also stimulate the direct production of *CCL18*, since this has been described in monocyte derived macrophages and primary alveolar macrophages [22]. Furthermore the release of *CCL18* in lepromatous lesions may lead to the development of more tolerant cells through a positive feedback loop [23], by promoting the differentiation of myeloid suppressor cells. These myeloid suppressor cells are thought to suppress the protective immune response in human T cells malignancies, and one could speculate that these cells could have a similar role in lepromatous leprosy. Furthermore, evidence that *CCL18* may play a direct role in leprosy is suggested by linkage and genome wide association studies that show several *CCL18* polymorphisms are associated with development of leprosy [33], [34]. In the GWAS study, three of eight *CCL18* polymorphisms were significantly associated with susceptibility to leprosy using a conventional significance threshold (but not GWAS level significance) [34]. Recent data suggest that *CCL18* is a ligand for *CCR8* and that it induces chemotaxis of TH2 polarized T cells [35]. Whether or not *CCL18* has primary effects on lepromatous leprosy development and persistence is currently unknown. However, these data suggest that *CCL18* may propagate lepromatous leprosy by recruiting TH2 T cells.

Our data show trends in levels of serum *CCL18* and *CCL17* that match clinical findings observed with mRNA levels in skin lesions and immunohistochemical analysis (for *CCL18*). Our study may be underpowered to determine a significant association between *CCL18* and *CCL17* serum levels and leprosy phenotype. Confirmation in larger studies will be needed to determine whether there is a correlation with increasing CCL18 and declining CCL17 levels in patients with lepromatous disease.

The association of *CCL17* and *CCL18* expression with younger individuals is an interesting result. While these data may suggest an influence of age on the dermal expression of these two cytokine, these data may also suggest an influence of the BCG vaccine that was uniformly administered by a nationwide program that began in Nepal in 1966. Our study did not record the vaccine status of the tested individuals. Further studies will be needed to determine whether expression of *CCL17* and *CCL18* is modulated by age or BCG vaccination.

There are several limitations to our study. First, a potential limitation is biopsy sampling error. We isolated RNA from biopsies that were adjacent to areas used to histologically classify the patient, and these two biopsies may have been significantly different. Although we cannot exclude that possibility, this limitation would apply to any leprosy biopsy study. Our patients were diagnosed comprehensively based upon slit skin smear, skin biopsy histopathology, clinical exam and neuropathy assessments; so there were unlikely to be large misclassifications due to biopsy sampling errors. Another possible limitation of this study is type II (false positive) error due to population bias. The population studied was recruited at a tertiary care center for leprosy, and contained a large percentage of individuals who were simultaneously undergoing Type I reversal and Type II ENL reactions. This bias may influence the types of cytokines and chemokines that are significantly different. Our analysis, however, showed that the main differences (*CCL17*, *CCL18*, and *CD14*) were preserved despite the presence or absence of reactive states in individuals.

Interestingly, our study demonstrated that *CCL17* and *CCL18* distinguished leprosy polarity with greater accuracy than the traditional TH1 and TH2 cytokines (*IL10*, *IFNG*). Although this difference may be due to a stronger biologic association of *CCL17* and *CCL18* with polarity, other explanations are also possible. First, many previous studies had small sample sizes and compared patients at the extreme end of the leprosy poles (for example, LL versus BT and TT) and did not include as many patients with the entire spectrum of leprosy as our study did. In addition, our samples did not proportionately represent the leprosy poles with many more LL (n = 27 or 61% of the lepromatous pole patients) than TT patients (n = 3, or 8% of the tuberculoid pole patients). These proportions could potentially skew the data in the favor of LL associations and weaken TT associations of traditional cytokines (*IFNG* with TH1 and tuberculoid leprosy).

## Supporting Information

Figure S1 **Correlation between Fluidigm gene chip analysis and Singleplex Real-Time PCR analysis.** Single assay probes for *CCL17* (A) and *CCL18* (B) that were normalized to GAPDH expression using standard RT-PCRa Fluidigmmicrofluidic platform. R^2^ values represent standard linear correlation.
(TIFF)

Table S1 **Primer-Probe sets used in this study.** A list of sequences of the primer and probe sets used in the paper. Probes were modified by 6-carboxyfluorescein (56-FAM) on the 5′ end and Iowa Black Quencher (3IABkFQ) on the 3′ end. The dyes also contain an additional internal quencher (ZEN).
(DOCX)
