# Supplementary figures and images for: Differential Dermal Expression of CCL17 and CCL18 in Tuberculoid and Lepromatous Leprosy
Source: PLoS Negl Trop Dis. 2014 Nov 20;8(11):e3263. doi: 10.1371/journal.pntd.0003263 (PMC4238987; doi:10.1371/journal.pntd.0003263)

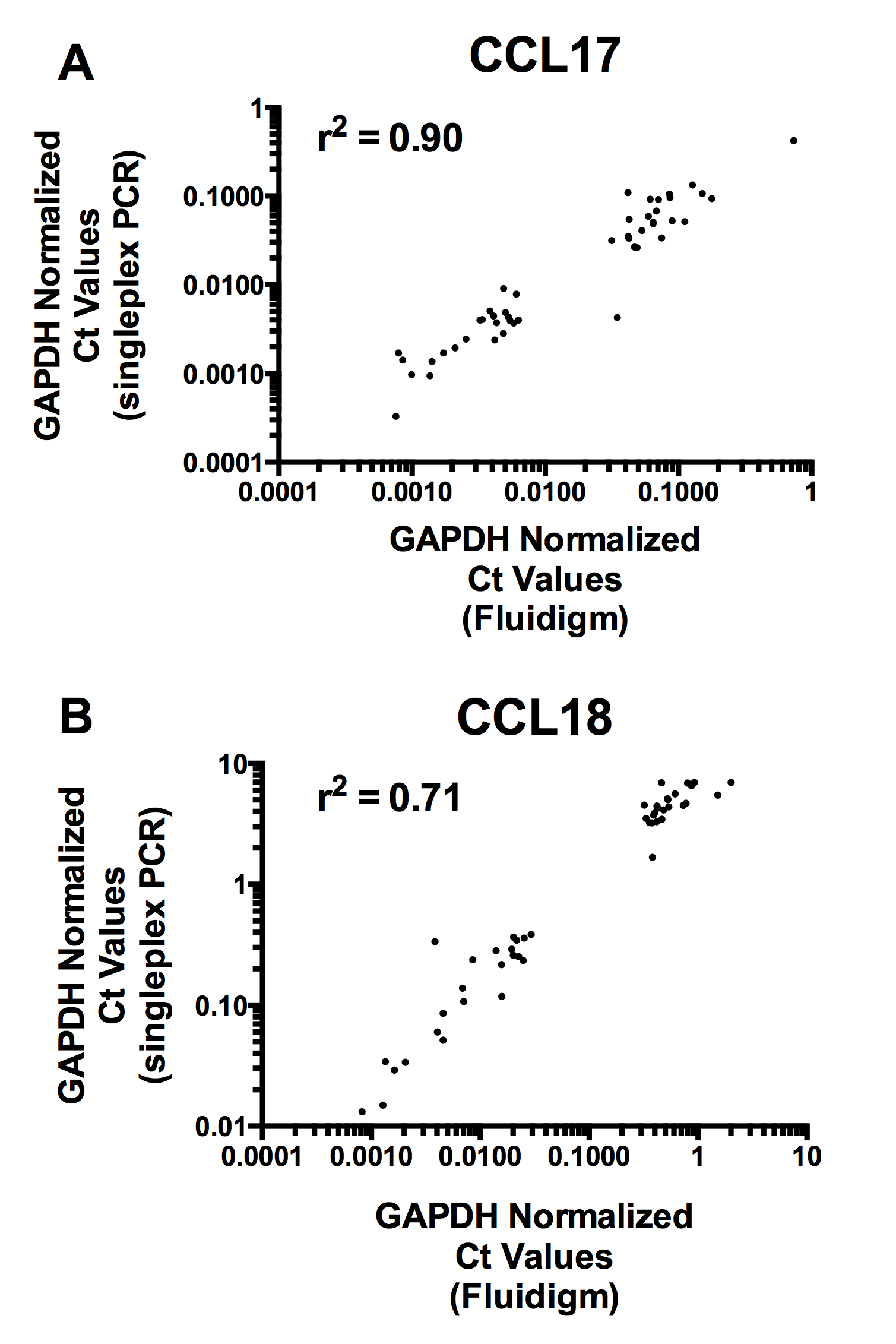

Supplement: Figure S1 — Correlation between Fluidigm gene chip analysis and Singleplex Real-Time PCR analysis. Single assay probes for CCL17 (A) and CCL18 (B) that were normalized to GAPDH expression using standard RT-PCRa Fluidigmmicrofluidic platform. R2 values represent standard linear correlation. (TIFF) [file pntd.0003263.s001.tiff]
