# Supplementary material for: Differential Dermal Expression of CCL17 and CCL18 in Tuberculoid and Lepromatous Leprosy
Source: PLoS Negl Trop Dis. 2014 Nov 20;8(11):e3263. doi: 10.1371/journal.pntd.0003263 (PMC4238987; doi:10.1371/journal.pntd.0003263)
Supplement: Table S1 — Primer-Probe sets used in this study. A list of sequences of the primer and probe sets used in the paper. Probes were modified by 6-carboxyfluorescein (56-FAM) on the 5′ end and Iowa Black Quencher (3IABkFQ) on the 3′ end. The dyes also contain an additional internal quencher (ZEN). (DOCX) [file pntd.0003263.s002.docx]

**Supplemental Table S1: Primer Probe sets used in this study.**

| **Gene** | **Group** | **Primer** | **Sequence** |
| --- | --- | --- | --- |
| CCL1 | Chemokine | Forward | CCG CAA ATG AGA AGC AAC ATC |
| CCL1 |  | Probe | /56-FAM/CGG AAG ATG /ZEN/TGG ACA GCA AGA GCA T/3IABkFQ/ |
| CCL1 |  | Reverse | CAG ACC AGA AGA CAT GCA GAT |
| CCL17 | Chemokine | Forward | CTG CAT TCT TCA CTC TCT TGT TG |
| CCL17 |  | Probe | /56-FAM/TGC TCC AGG /ZEN/GAT GCC ATC GTT /3IABkFQ/ |
| CCL17 |  | Reverse | CTT AGA AAG CTG AAG ACG TGG T |
| CCL18 | Chemokine | Forward | ACT ATG AAC TTT TGT GGA ATC TGC |
| CCL18 |  | Probe | /56-FAM/AGC TCT GCT /ZEN/GCC TCG TCT ATA CCT /3IABkFQ/ |
| CCL18 |  | Reverse | CTC CTT GTC CTC GTC TGC |
| CCL2 | Chemokine | Forward | CAC TGA GAT CTT CCT ATT GGT GA |
| CCL2 |  | Probe | /56-FAM/CGC TCA GCC /ZEN/AGA TGC AAT CAA TGC /3IABkFQ/ |
| CCL2 |  | Reverse | TCA TAG CAG CCA CCT TCA TTC |
| CD14 | Cell Marker | Forward | AAT CTT CAT CGT CCA GCT CAC |
| CD14 |  | Probe | /56-FAM/CGC AGA GAC /ZEN/GTG CAC CAG C/3IABkFQ/ |
| CD14 |  | Reverse | CAG AGG TTC GGA AGA CTT ATC G |
| CD209 | Cell Marker | Forward | GCT CTC CTC TGT TCC AAT ACT G |
| CD209 |  | Probe | /56-FAM/TCA CCT CTG /ZEN/TTG CCC AGC TTC AA/3IABkFQ/ |
| CD209 |  | Reverse | CTT CAC CTG GAT GGG ACT TTC |
| CD22 | Cell Marker | Forward | GAG ATG CAT GGT GTC GTG T |
| CD22 |  | Probe | /56-FAM/CCT GTT TCC /ZEN/GCG TGT CTT C CC T/3IABkFQ/ |
| CD22 |  | Reverse | CTC CTT TTG CTC TCA GAT GCT |
| CD3D | Cell Marker | Forward | TCA CAA ACA CTC TGT CCT CAA G |
| CD3D |  | Probe | /56-FAM/TCG CAA GTG /ZEN/AGC CCC TTC AAG ATA C/3IABkFQ/ |
| CD3D |  | Reverse | GGA GAT GGA ACA TAG CAC GTT |
| FOXP3 | Cell Marker | Forward | CAT TGA GTG TCC GCT GCT |
| FOXP3 |  | Probe | /56-FAM/CCT TTC ACC /ZEN/TAC GCC ACG CTC AT/3IABkFQ/ |
| FOXP3 |  | Reverse | GCA CAT TCC CAG AGT TCC T |
| GAPDH | House  Keeping | Forward | GGC CAT CCA CAG TCT TCT G |
| GAPDH |  | Probe | /56-FAM/ATG ACC ACA /ZEN/GTC CAT GCC ATC ACT /3IABkFQ/ |
| GAPDH |  | Reverse | CAG CCT CAA GAT CAT CAG CAA |
| IFNA1 | Interferon | Forward | TCA GCA TGG TCA TAG TTA TAG CAG |
| IFNA1 |  | Probe | /56-FAM/ACA GAA TTC /ZEN/ATG AAA GCG TGA CCT GGT /3IABkFQ/ |
| IFNA1 |  | Reverse | GCA AGA AAG ATT AAG GAG GAA GGA A |
| IFNA8 | Interferon | Forward | TCA TAG CAT GGT TTT GGC AGA |
| IFNA8 |  | Probe | /56-FAM/ACA GCA GCT /ZEN/CAC ACT TCG ACA AGT /3IABkFQ/ |
| IFNA8 |  | Reverse | AGA GTA AGG AAT GAG ACC TGG T |
| IFNB1 | Interferon | Forward | GCA GTA CAT TAG CCA TCA GTC A |
| IFNB1 |  | Probe | /56-FAM/TGA AGC AAT /ZEN/TGT CCA GTC CCA GAG G/3IABkFQ/ |
| IFNB1 |  | Reverse | ACT GAA GAT CTC CTA GCC TGT |
| IFNG | Interferon | Forward | CGA CAG TTC AGC CAT CAC TT |
| IFNG |  | Probe | /56-FAM/TCG GTA ACT /ZEN/GAC TTG AAT GTC CAA CGC /3IABkFQ/ |
| IFNG |  | Reverse | GCA ACA AAA AGA AAC GAG ATG AC |
| IL10 | Cytokine | Forward | CTG GAT CAT CTC AGA CAA GGC |
| IL10 |  | Probe | /56-FAM/TGG AGG ACT /ZEN/TTA AGG GTT ACC TGG GT/3IABkFQ/ |
| IL10 |  | Reverse | TCA AAT GAA GGA TCA GCT GGA C |
| IL12A | IL12 Class | Forward | CCA CCT GGT ACA TCT TCA AGT C |
| IL12A |  | Probe | /56-FAM/AAC TAA TGG /ZEN/GAG TTG CCT GGC CT/3IABkFQ/ |
| IL12A |  | Reverse | ACC AAG AAT GAG AGT TGC CTA A |
| IL12B | IL12 Class | Forward | CTG AGG TCT TGT CCG TGA AG |
| IL12B |  | Probe | /56-FAM/TGA CAT TCT /ZEN/GCG TTC AGG TCC AGG /3IABkFQ/ |
| IL12B |  | Reverse | GTA CTC CAC ATT CCT ACT TCT CC |
| IL13 | Cytokine | Forward | TGA TGC TCC ATA CCA TGC TG |
| IL13 |  | Probe | /56-FAM/CAG AAC CAG /ZEN/AAG GCT CCG CTC T/3IABkFQ/ |
| IL13 |  | Reverse | GTC ATT GCT CTC ACT TGC CT |
| IL17A | T cell cytokine | Forward | CAC TTT GCC TCC CAG ATC AC |
| IL17A |  | Probe | /56-FAM/CAC CGC AAT /ZEN/GAG GAC CCT GAG A/3IABkFQ/ |
| IL17A |  | Reverse | ACT ACA ACC GAT CCA CCT CA |
| IL18 | IL1b Class | Forward | CAC AGA GAT AGT TAC AGC CAT ACC |
| IL18 |  | Probe | /56-FAM/TGT AGA GAT /ZEN/AAT GCA CCC CGG ACC A/3IABkFQ/ |
| IL18 |  | Reverse | TCG GCC TCT ATT TGA AGA TAT GAC |
| IL1B | IL1b Class | Forward | GTC ATC CTC ATT GCC ACT GTA |
| IL1B |  | Probe | /56-FAM/AGA AGT ACC /ZEN/TGA GCT CGC CAG TGA /3IABkFQ/ |
| IL1B |  | Reverse | CAG CCA ATC TTC ATT GCT CAA G |
| IL1RN | IL1b Class | Forward | TTG TCC TGC TTT CTG TTC TCG |
| IL1RN |  | Probe | /56-FAM/TCA GTG ATG /ZEN/TTA ACT GCC TCC AGC TG/3IABkFQ/ |
| IL1RN |  | Reverse | CTG TCC TGT GTC AAG TCT GG |
| IL21 | Cytokine | Forward | GTT CTA GAG GAC AGA TGC TGA TG |
| IL21 |  | Probe | /56-FAM/TCC TAG AAA /ZEN/GAT TCA AAT CAC TTC TCC AAA AGA TGA /3IABkFQ/ |
| IL21 |  | Reverse | GAC AGA AAC ACA GAC TAA CAT GC |
| IL22 | Cytokine | Forward | CTC TGG ATA TGC AGG TCA TCA C |
| IL22 |  | Probe | /56-FAM/TGA CAT GTG /ZEN/CTT AGC CTG TTG CTG A/3IABkFQ/ |
| IL22 |  | Reverse | AGT GCT GTT CCC TCA ATC TG |
| IL23A | IL12 Class | Forward | GAT CCT TTG CAA GCA GAA CTG |
| IL23A |  | Probe | /56-FAM/CCA GTG TGG /ZEN/AGA TGG CTG TGA CC/3IABkFQ/ |
| IL23A |  | Reverse | AGA GGG AGA TGA AGA GAC TAC A |
| IL27 | IL12 Class | Forward | GCT GCA TCC TCT CCA TGT TG |
| IL27 |  | Probe | /56-FAM/TCT GCT TCA /ZEN/TCT CCA CCA CGC TTC /3IABkFQ/ |
| IL27 |  | Reverse | CTG ATG TTT CCC TGA CCT TCC |
| IL-29 (IFNL1) | Interferon | Forward | GAA GAC AGG AGA GCT GCA AC |
| IL-29 (IFNL1) |  | Probe | /56-FAM/TTG AGT GAC /ZEN/TCT TCC AAG GCG TCC /3IABkFQ/ |
| IL-29 (IFNL1) |  | Reverse | GGC AGG TTC AAA TCT CTG TCA |
| IL4 | Cytokine | Forward | CTC ATG GTG GCT GTA GAA CTG |
| IL4 |  | Probe | /56-FAM/TCC AAG AAC /ZEN/ACA ACT GAG AAG GAA ACC TT/3IABkFQ/ |
| IL4 |  | Reverse | CCT CAC AGA GCA GAA GAC TC |
| IL6 | Cytokine | Forward | CTC TTT GCT GCT TTC ACA CAT |
| IL6 |  | Probe | /56-FAM/AGA TGC CGT /ZEN/CGA GGA TGT ACC GA/3IABkFQ/ |
| IL6 |  | Reverse | CAC TCA CCT CTT CAG AAC GAA T |
| TNF | Cytokine | Forward | TTC GAG AAG ATG ATC TGA CTG C |
| TNF |  | Probe | /56-FAM/CGC CAC CAC /ZEN/GCT CTT CTG C/3IABkFQ/ |
| TNF |  | Reverse | AGC CTC TTC TCC TTC CTG AT |
